# Supplementary material for: Key Factors in Enhancing Pseudocapacitive Properties of PANI-InOx Hybrid Thin Films Prepared by Sequential Infiltration Synthesis
Source: Polymers (Basel). 2023 Jun 8;15(12):2616. doi: 10.3390/polym15122616 (PMC10300785; doi:10.3390/polym15122616)
Supplement: Supplementary file 1 [file polymers-15-02616-s001.zip › polymers-2419154-supplementary.pdf]

Supporting Information

Key Factors in Enhancing Pseudocapacitive Properties of PANI-InO_x Hybrid Thin Films Prepared by Sequential Infiltration Synthesis

Jiwoong Ham [1], Hyeong-U Kim [2] and Nari Jeon [1,*]

[1] Department of Materials Science and Engineering, Chungnam National University, Daejeon 34134, Republic of Korea; maxbasic55@o.cnu.ac.kr
[2] Department of Plasma Engineering, Korea Institute of Machinery & Materials (KIMM), Daejeon 34103, Republic of Korea; guddn418@kimm.re.kr
* Corresponding author: njeon@cnu.ac.kr

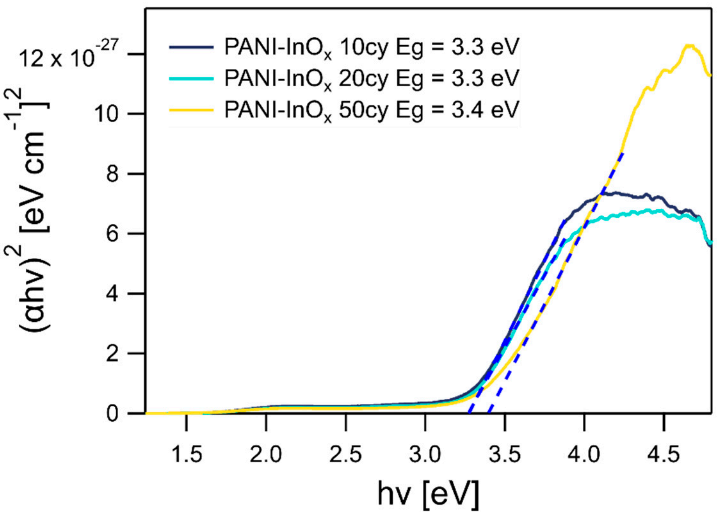

Figure S1. Tauc plots of PANI-InO_x sample prepared with 10, 20, and 50 SIS cycles.

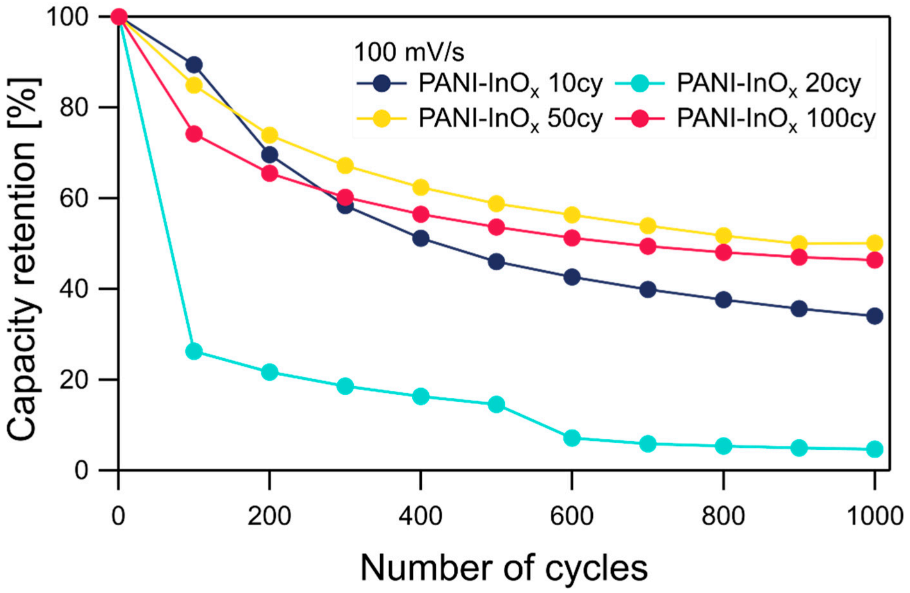

Figure S2. Capacity retention test of PANI-InO_x samples prepared with 10, 20, 50, and 100 SIS cycles. The CVs were collected at a scan rate of 100 mV/sec in an aqueous electrolyte of neutral pH.

Capacitance retention tests (**Figure S2**) were performed for 1,000 cycles at a scan rate of 100 mV/s within the potential window, as shown in **Figure 4**. The samples were exposed to the electrolyte for approximately 6 h. Most samples, except for the 20 cy sample displayed a gradual decrease in capacitance values. This decreasing trend can be attributed to the dissolution of composite films in the electrolyte[1]. The 50 cy sample, which had the highest capacitance value among the test samples, exhibited the highest capacitance retention.

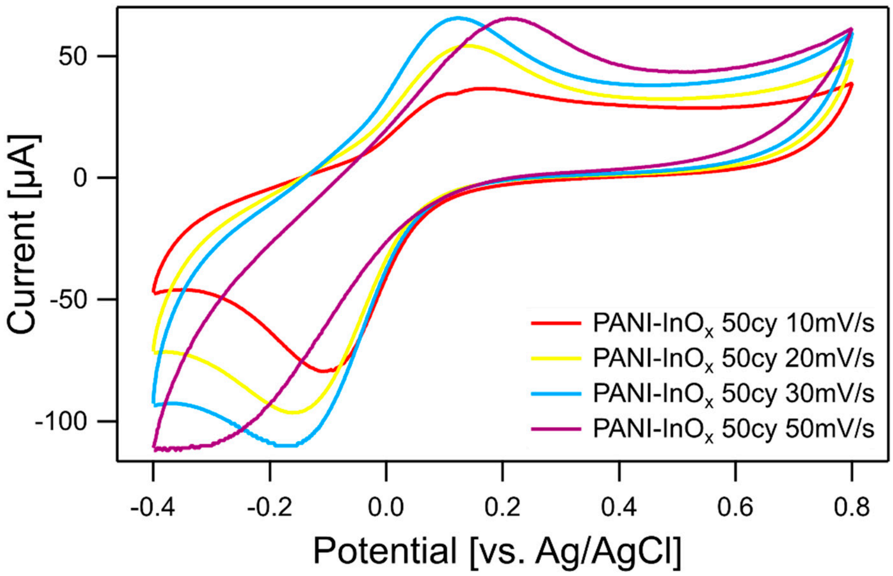

Figure S3. CV curves of 50 cy PANI-InO_x sample collected at different scan rates as noted in the Figure.

Reference

(1) Yue, J.; Lin, L.; Jiang, L.; Zhang, Q.; Tong, Y.; Suo, L.; Hu, Y.-s.; Li, H.; Huang, X.; Chen, L. Interface Concentrated-Confinement Suppressing Cathode Dissolution in Water-in-Salt Electrolyte. *Adv. Energy Mater.* **2020**, *10* (36), 2000665.https://doi.org/10.1002/aenm.202000665
